# Interpolation Methods with Phase Control for Backprojection of Complex-Valued SAR Data †

**DOI:** 10.3390/s22134941

**Published:** 2022-06-30

**Authors:** Yevhen Ivanenko, Viet T. Vu, Aman Batra, Thomas Kaiser, Mats I. Pettersson

**Affiliations:** 1 Department of Mathematics and Natural Sciences, Blekinge Institute of Technology, 371 79 Karlskrona, Sweden; viet.thuy.vu@bth.se (V.T.V.); mats.pettersson@bth.se (M.I.P.); 2 Institute of Digital Signal Processing, University of Duisburg-Essen, 47057 Duisburg, Germany; aman.batra@uni-due.de (A.B.); thomas.kaiser@uni-due.de (T.K.)

**Keywords:** complex-valued SAR data interpolation, complex SAR interpolation, THz SAR, backprojection, GBP

## Abstract

Time-domain backprojection algorithms are widely used in state-of-the-art synthetic aperture radar (SAR) imaging systems that are designed for applications where motion error compensation is required. These algorithms include an interpolation procedure, under which an unknown SAR range-compressed data parameter is estimated based on complex-valued SAR data samples and backprojected into a defined image plane. However, the phase of complex-valued SAR parameters estimated based on existing interpolators does not contain correct information about the range distance between the SAR imaging system and the given point of space in a defined image plane, which affects the quality of reconstructed SAR scenes. Thus, a phase-control procedure is required. This paper introduces extensions of existing linear, cubic, and sinc interpolation algorithms to interpolate complex-valued SAR data, where the phase of the interpolated SAR data value is controlled through the assigned a priori known range time that is needed for a signal to reach the given point of the defined image plane and return back. The efficiency of the extended algorithms is tested at the Nyquist rate on simulated and real data at THz frequencies and compared with existing algorithms. In comparison to the widely used nearest-neighbor interpolation algorithm, the proposed extended algorithms are beneficial from the lower computational complexity perspective, which is directly related to the offering of smaller memory requirements for SAR image reconstruction at THz frequencies.

## 1. Introduction

Synthetic aperture radar (SAR) is a remote sensing technique that has been developed since the 1950s as an alternative to the optical imaging system and with the aim to acquire images with a high cross-range resolution. Nowadays, it is used in a wide range of applications, including stationary and moving target detection [1,2].

The range of SAR applications is directly correlated to the frequency band of the SAR systems. For example, SAR systems, such as spaceborne and airborne, commonly operate in the microwave frequency range below 30 GHz. The spaceborne SAR systems, including TerraSAR-X, RADARSAT-2, and COSMO-SkyMed, provide an opportunity to study geoscience and hydrology at a distance of hundred kilometers from the Earth’s surface [3]. The airborne SARs that operate in this frequency range include ultrawideband-ultrawidebeam SAR imaging systems (UWB SAR) that have been developed to perform high-resolution ground imaging, even under adverse weather conditions [4]. The UWB SARs refer to the systems that utilize radar signals with large fractional bandwidth and synthesize a wide integration angle [4,5]. The typical examples of UWB SAR systems are CARABAS operating in the frequency range [20, 90] MHz[6] and LORA that operates in the frequency range [200, 800] MHz [7].

The THz frequency spectrum is also suitable to achieve high-resolution imaging. The SAR systems that operate at these frequencies are suitable for short-range applications, including the indoor environment purpose. The development of such type of systems is quite an active research topic, which has potential applications in the areas of security, logistics, and medicine. Most of the state-of-the-art THz SARs have been designed to process bandpass signals and are realized as ground- and rail-based systems. For example, in [8,9,10], SAR imaging is performed at 0.3THz, and, in [11,12], the imaging has been implemented at frequencies 0.6THz and 0.75THz, respectively. Recent results include imaging at 1.1THz that have been published in [13].

Nevertheless, modern SAR systems face different technical issues caused by the deviation of SAR platforms from the path. For example, unmanned aerial vehicle (UAV) SAR imaging systems deal with platform vibrations. This problem becomes crucial for UAV-based SARs that operate in the THz frequency range, which are extremely sensitive to the platform path deviation [14,15]. To deal with this issue, the use of signal processing algorithms that are capable to handle motion error compensation is required.

Among the SAR image formation algorithms, the backprojection algorithms, such as Global Backprojection (GBP) [16], Fast Backprojection [17], and Fast Factorized Backprojection [18], have their own capability to manage the motion error compensation [19] based on a priori knowledge on SAR platform deviation from the expected path [20]. Due to the fact that in these algorithms the backprojection procedure always requires range calculation for each aperture position, information about the platform deviations can be included in the backprojection algorithm as a motion compensation procedure at this stage. In addition, the backprojection algorithms are capable to handle radar signals with a large fractional bandwidth [5,19]. For these reasons, the backprojection algorithms play an important role in SAR data processing despite their high computational cost. One of the contributions to the high computational cost is the interpolation procedure, at which a complex data value is estimated based on given complex SAR data values for a given range-time delay. Then, the interpolated complex value is backprojected into the defined image plane.

There exist various interpolation algorithms that are used for SAR applications. Hanssen and Bamler introduced an evaluation of nearest neighbor, piecewise linear, four- and six-point cubic convolution, and truncated sinc interpolators in [21] and presented their application to SAR interferometry. According to the evaluation [21], the four-point cubic convolution interpolator has been considered as the optimal interpolator among the described interpolation algorithms. However, for high-resolution applications, the six-point cubic convolution interpolator has been recommended. To improve coherency in SAR interferometry, it has been proposed to combine the truncated sinc interpolator with the Hanning window [22]. Nevertheless, the results obtained in [22] have demonstrated that there is no interpolator for SAR image resampling that is optimal for all SAR data types and quality. Capazzoli et al. have introduced in [23] Knab and approximate prolate windows for sinc interpolator that is used for SAR backprojection and compared their efficiency in terms of the root mean square error (RMSE). The results demonstrate that windowed sinc interpolators provide better accuracy (the RMSE is about 0.01%) than the other interpolators.

To the knowledge of the authors, none of the interpolators described above, with the exception of the nearest neighbor, the performance of which can be improved through the support of the FFT interpolation [20], has been clearly formulated to interpolate SAR data with complex-valued representation. The FFT interpolation in combination with the nearest neighbor interpolator can lead to a huge amount of unused data, which might not be stored or processed by the real-time THz SAR system due to constructive limitations (e.g., installation of such a system on compact UAV platforms to monitor the environment, which brings limitations on energy and computational power). Furthermore, none of these interpolators accounts for the relationship between the range distance from the SAR platform antenna to the point in the defined image plane and the phase of interpolated SAR data value, which affects the accuracy of reconstructed SAR scenes. In the rest of the paper, we define SAR data with complex-valued representation as complex SAR data.

In this paper, we introduce the extended versions of linear, cubic, and sinc interpolation algorithms that, in comparison with the interpolators described in [21,22,23], include the phase control procedure. The initial results on the phase control of complex SAR data under linear and cubic interpolation have been partially reported in [24]. The idea of the proposed control procedure is naturally related to the complex SAR data processing. To control the argument of the interpolated SAR data value, a priori known information about the range distance between the SAR platform antenna and the given point in the defined image plane has to be assigned to the phase of surrounding nearest neighbor sample points. The procedure is implemented through the multiplication of surrounding nearest neighbor data samples with corresponding phase compensation terms. The developed interpolators can be incorporated into the backprojection algorithms to process complex SAR data, and GBP is selected as an example in this paper. The results achieved with the developed interpolators are compared with the results obtained with the nearest neighbor and corresponding conventional (existing) interpolation algorithms. Furthermore, the efficiency of the developed interpolators is tested on simulated and real data at THz frequencies, where the accuracy of interpolation methods plays a crucial role, and is verified by comparison with the results provided by the analytical approach introduced in [4]. The effects of the sampling rate on the accuracy of SAR image formation are also considered in this paper. The novelty of the proposed scheme is highly beneficial for SAR sensing at THz frequencies. The computational complexity drastically increases at the THz spectrum due to the large number of pixels to be processed. Especially for UAV-based THz SARs, the payload and energy resources are limited and hence the limited computational power for on-board processing.

The rest of the paper is organized as follows. Section 2 describes the problem formulation, which includes the measurement setup description, the image formation process, and the proposed phase-control procedure. In Section 3, the extended versions of the existing interpolation algorithms, which are adopted for processing complex SAR data, are described. The simulation-based examples are considered to study the efficiency of the extended interpolation algorithms in Section 4. The practical application of the developed interpolation algorithms in the processing of the data that has been acquired at THz frequencies is presented in Section 5. The paper is concluded in Section 6. Furthermore, the impulse response function for SAR scenes that contain a point target in their center is described in Appendix A and the peak-sidelobe ratio is introduced in Appendix B.

## 2. Problem Formulation

In this section, we introduce a measurement setup and the image formation process based on the time-domain GBP algorithm. The novel part of the image-formation process is the phase control of estimated complex SAR data, which is performed under the interpolation stage of the backprojection algorithm to assign the range distance between the SAR imaging system and the given point of space in the defined image plane.

### 2.1. Notation and Conventions

Throughout this paper, the time convention $e^{j2\pi ft}$ is used for raw data, where *f* denotes the frequency and *t* the time. Let $\mu_{0}$, $\epsilon_{0}$, and $c_{0}$ denote the permeability, the permittivity, and the speed of light in vacuum, respectively, where $c_{0}=1/\sqrt{\mu_{0}\epsilon_{0}}$. The real, the imaginary parts, and the complex conjugate of a complex number ζ, ζ∈C, are denoted by Re{ζ}, Im{ζ}, and $\zeta^{*}$, respectively. Finally, the right and the left complex half-planes are indicated by $C_{+}$ and $C_{-}$, respectively, where C+={ζ∈C|Re{ζ}>0} and C−={ζ∈C|Re{ζ}<0}, respectively.

### 2.2. Setup

**Figure 1:**
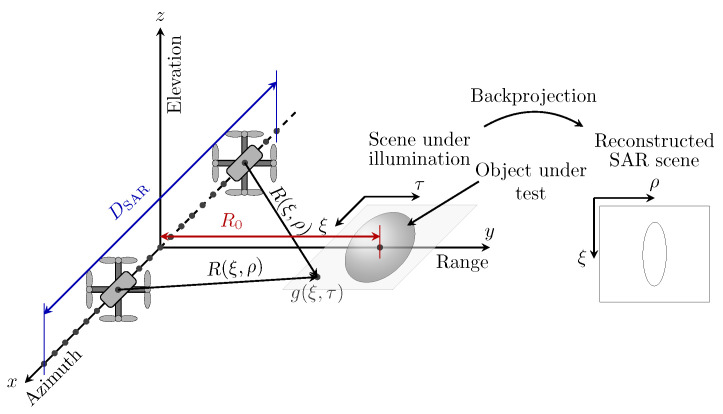
Problem setup. Representation of a UAV-based THz SAR imaging system. Here, $R_{0}$ denotes the reference (minimal) range distance between the platform antenna and the center of the object located in the scene under illumination.

Consider a monostatic UAV-based SAR imaging system that transmits frequency modulated signals $s_{t}\left(\tau \right)$ and receives backscattered echoes of a similar waveform $s_{r}\left(\xi ,\tau \right)$ at the same aperture position, where τ denotes the range time and ξ the azimuth. The SAR system operates at THz frequencies and is mounted on a quadcopter, which is one of the desirable platforms for such systems under development; see the problem setup in Figure 1.

Assume that the THz SAR system follows the straight path that agrees with the azimuth axis, and the object under test is of an arbitrary shape and located in the center of the scene under illumination at the reference range distance $R_{0}$. The range distance between the platform antenna and the point of the scene under illumination at each aperture position can be defined as

(1) $$R=\sqrt{{\left(\xi -\xi^{\prime }\right)}^{2}+{\left(\rho^{\prime }\right)}^{2}},$$

where (ξ,0) and $\left(\xi^{\prime },\rho^{\prime }\right)$ are the coordinates of the actual aperture position and the point of the scene under illumination, respectively.

### 2.3. Image Formation

The range-compressed received signal $s_{r}\left(\xi ,\tau \right)$ can be obtained through the matched-filtering procedure, i.e., $g=s_{r}\left(\xi ,\tau \right)*s_{t}^{*}\left(-\tau \right)$, where ∗ denotes the convolution operator. Let GBP be the algorithm to be used to form the SAR image from the raw data g(ξ,τ). Furthermore, let the slant-range plane be the image plane, into which the raw data *g* is backprojected. Then, a SAR image h(ξ,ρ) can be formed through the superposition of acquired data g(ξ,τ) and its corresponding backprojection into the image plane, which is expressed mathematically as

(2) $$h\left(\xi ,\rho \right)=\int_{-D_{\mathrm{SAR}}/2}^{D_{\mathrm{SAR}}/2}g\left(\xi ,\tau \right)d\;\xi ,$$

where $D_{\mathrm{SAR}}$ is the length of synthetic aperture [25]. This expression is applied to common pulse radars that use chirp signals.

The backprojection algorithm (2) can also be applied to other types of data with small modifications. For example, for a FMCW radar, the Fourier transform in the range direction is required to be applied before the backprojection process. Radar measurements can also be based on a vector network analyzer (VNA), in which the output is the reflection coefficient $S_{11}$. The measured data in the time domain can then be considered based on the Born approximation as a range-compressed signal from multiple point targets, which is expressed analytically as

(3) $$g\left(\xi ,\tau \right)\approx \sum_{j=1}^{K}A_{tj}\mathrm{sinc}\left[\pi \left(f_{\max}-f_{\min}\right)\left(\tau -\tau_{tj}\right)\right]e^{j2\pi f_{c}\left(\tau -\tau_{tj}\right)},$$

where $f_{\min}$ and $f_{\max}$ are the lowest and the highest frequencies processed, respectively, f≥0, $A_{tj}$ is the backscattering amplitude from the corresponding *j*-th target, $f_{c}=\left(f_{\max}+f_{\min}\right)/2$ the center frequency, and $\tau_{tj}$ the two range time, i.e., the time required for the wave to travel from antenna to the corresponding *j*-th target and backwards. The reflection coefficient peaks occur at the radar ranges, where targets are present. For this reason, the backprojection process (2) can be directly applied to the output of VNA.

The fast and fast factorized backprojection algorithms operate similarly to GBP. However, in these algorithms, the acquired data g(ξ,τ) are divided into subsets (subapertures) along the azimuth axis. Each of the subsets is then superposed separately before backprojection into the corresponding sub-image plane to form the final image.

### 2.4. Phase Control of Estimated Complex SAR Data

The interpolation procedure is required by the GBP algorithm, which is described in Section 2.3. To ensure that the data is backprojected correctly, a phase control under interpolation is necessary.

Let $\tau_{p}$ denote the two range traveling time from the aperture position (ξ,0) to the image position $\left(\xi_{p},\rho_{p}\right)$ given by

(4) $$\tau_{p}=\frac{2}{c_{0}}\sqrt{{\left(\xi -\xi_{p}\right)}^{2}+\rho_{p}^{2}}.$$

In modern radar systems, the raw data g(ξ,τ) is sampled signal in time domain. To get the corresponding complex value at the range time $\tau_{p}$, surrounding samples of *g* given as a function of range time $\tau_{i}$ for fixed azimuth ξ have to be used for reconstruction, which are of the form

(5) $$g\left(\xi ,\tau_{i}\right)\approx \sum_{j=1}^{K}A_{tj}\mathrm{sinc}\left[\pi \left(f_{\max}-f_{\min}\right)\left(\tau_{i}-\tau_{tj}\right)\right]e^{j2\pi f_{c}\left(\tau_{i}-\tau_{tj}\right)}.$$

Here, i=0,…,N, where the number of samples *N* depends on the one of the interpolation methods that will be presented in Section 3. Note that the information about the range distance between the SAR system and the target is contained in the phase of complex SAR data, and thus phase control under the estimation of $g(\xi ,\tau_{p})$ is required. To achieve this, the following compensation procedure is introduced

(6) $$\tilde{g}\left(\xi ,\tau_{i}\right)=g\left(\xi ,\tau_{i}\right)e^{j2\pi f_{c}\left(\tau_{p}-\tau_{i}\right)}\approx \sum_{j=1}^{K}A_{tj}\mathrm{sinc}\left[\pi \left(f_{\max}-f_{\min}\right)\left(\tau_{i}-\tau_{tj}\right)\right]e^{j2\pi f_{c}\left(\tau_{p}-\tau_{tj}\right)},$$

which is only used under the local interpolation procedure to estimate $g(\xi ,\tau_{p})$. Here, the proposed procedure assigns the information about the range time difference between the pixel in the defined image plane and the target to the samples to be used in the interpolation procedure. Correspondingly, the interpolated parameter $g(\xi ,\tau_{p})$ will contain the assigned phase information. Interpolation algorithms that include the proposed phase-control procedure (6) will be described in Section 3.

## 3. Interpolation Methods

In this section, a set of methods for interpolation of complex SAR data is introduced. Note that all the described methods, with the exception of nearest-neighbor interpolation, involve the phase-control procedure that takes an important place in the processing of complex SAR data. The proposed interpolation methods can further be incorporated into different backprojection algorithms.

### 3.1. Nearest Neighbor Interpolation

Nearest neighbor interpolation is one of the simplest interpolation methods, the principle of which is to estimate a value of the unknown sample by assigning the known data value of the nearest neighbor sample. The nearest neighbor interpolator $p_{n}\left(\tau \right)$ can be described on the real-valued interval $\left[\tau_{0},\tau_{1}\right]$, $\tau_{0}<\tau_{1}$, by the following relation

(7) $$p_{n}\left(\tau \right)=\left\{\begin{array}{c} y_{0},\;\tau_{0}\leq \tau <\frac{1}{2}\left(\tau_{0}+\tau_{1}\right),\;\\ y_{1},\;\frac{1}{2}\left(\tau_{0}+\tau_{1}\right)<\tau \leq \tau_{1}. \end{array}\right.$$

Here, $y_{0},y_{1}\in C$ denote the known data samples.

### 3.2. Extended Linear-Spline Interpolation

Linear spline function for complex-valued data interpolation $p_{l}\left(\tau \right)$ is a linear polynomial function on the interval $\left[\tau_{0},\tau_{1}\right]$, τ∈R, where the samples $\tau_{0}<\tau_{1}$ are the knot points. The linear spline $p_{l}$ is uniquely defined by

(8) $$p_{l}\left(\tau \right)=a+b\left(\tau -\tau_{0}\right),\;\tau_{0}\leq \tau \leq \tau_{1},$$

with the corresponding derivative

(9) $$p_{l}^{\prime }\left(\tau \right)=b,\;\tau_{0}\leq \tau \leq \tau_{1},$$

and where a,b∈C are the polynomial coefficients. Furthermore, the spline $p_{l}$ satisfies the following spline conditions

(10) $$\left\{\begin{array}{c} p_{l}\left(\tau_{0}\right)={\tilde{y}}_{0},\;\\ p_{l}\left(\tau_{1}\right)={\tilde{y}}_{1}, \end{array}\right.$$

where ${\tilde{y}}_{i}$, i=0,1, are the data values, the phase of which is controlled through the procedure (6). It should be noted that the phase controlled in ${\tilde{y}}_{i}$ may vary with a factor of π due to the corresponding location of complex data parameters either in the right $C_{+}$ or in the left complex half-plane $C_{-}$.

Assume that the range time $\tau_{p}$ defined in (4) satisfies the condition $\tau_{0}<\tau_{p}<\tau_{1}$. Then, by employing the spline conditions (10), the following system of equations can be constructed

(11) Ac=y,

where

(12) $$A=\left[\begin{array}{cc} 1 & 0\;\\ 1 & \tau_{1}-\tau_{0} \end{array}\right],$$

$c={\left[a\;b\right]}^{T}$, and $y={\left[{\tilde{y}}_{0}\;{\tilde{y}}_{1}\right]}^{T}$. The resulting filter that provides complex-valued data estimation at range time $\tau_{p}$ based on linear-spline interpolation with phase control is given by

(13) $$p_{l}\left(\tau_{p}\right)=a+b\left(\tau_{p}-\tau_{0}\right).$$

Here, the polynomial coefficients $a={\tilde{y}}_{0}$ and $b=\left({\tilde{y}}_{1}-{\tilde{y}}_{0}\right)/\left(\tau_{1}-\tau_{0}\right)$ are determined from the system of equations (11).

### 3.3. Extended Cubic-Spline Interpolation

Cubic spline function for complex-valued data interpolation $p_{c}\left(\tau \right)$ is a piecewise cubic polynomial function on the interval $\left[\tau_{0},\tau_{2}\right]$, τ∈R, and where the samples $\tau_{i-1}<\tau_{i}$ for i=1,2 are the knot points. The cubic spline $p_{c}$ can be uniquely defined by

(14) $$p_{c,i}\left(\tau \right)=a_{i}+b_{i}\left(\tau -\tau_{i-1}\right)+c_{i}{\left(\tau -\tau_{i-1}\right)}^{2}+d_{i}{\left(\tau -\tau_{i-1}\right)}^{3},\;\tau_{i-1}\leq \tau \leq \tau_{i},$$

with corresponding first

(15) $$p_{c,i}^{\prime }\left(\tau \right)=b_{i}+2c_{i}\left(\tau -\tau_{i-1}\right)+3d_{i}{\left(\tau -\tau_{i-1}\right)}^{2},\;\tau_{i-1}\leq \tau \leq \tau_{i},$$

second

(16) $$p_{c,i}^{\prime \prime }\left(\tau \right)=2c_{i}+6d_{i}\left(\tau -\tau_{i-1}\right),\;\tau_{i-1}\leq \tau \leq \tau_{i},$$

and third derivatives

(17) $$p_{c,i}^{\prime \prime \prime }\left(\tau \right)=6d_{i},\;\tau_{i-1}\leq \tau \leq \tau_{i}.$$

Here, $a_{i},b_{i},c_{i},d_{i}\in C$ for i=1,2 are the polynomial coefficients. Furthermore, the spline function satisfies the following conditions

(18) $$\left\{\begin{array}{c} p_{c,i}\left(\tau_{i-1}\right)={\tilde{y}}_{i-1},\;\\ p_{c,i}\left(\tau_{i}\right)={\tilde{y}}_{i},\;\\ p_{c,i}^{\prime }\left(\tau_{i}\right)=p_{c,i+1}^{\prime }\left(\tau_{i}\right),\;\\ p_{c,i}^{\prime \prime }\left(\tau_{i}\right)=p_{c,i+1}^{\prime \prime }\left(\tau_{i}\right) \end{array}\right.$$

for i=1,2, and where the last two conditions are not applicable at the edge knot points $\tau_{0}$ and $\tau_{2}$. Here, $\tilde{y}$ denotes the reference data values, the phase of which is controlled under the procedure introduced in (6). It should be noted that controlled phase value may vary with a factor of π due to corresponding location of data values in the complex plane, i.e., either in the right $C_{+}$ or in the left complex half-plane $C_{-}$.

Assume that the range time $\tau_{p}$ defined in (4) satisfies the condition $\tau_{0}<\tau_{p}<\tau_{1}$. Let $p_{c,i}^{\prime \prime }\left(\tau_{i-1}\right)=k_{i-1}$, from which the unknown polynomial coefficient can be expressed as $c_{i}=k_{i-1}/2$. Furthermore, let $\delta_{i-1}=\tau_{i}-\tau_{i-1}$. By employing spline conditions (18), the unknown polynomial coefficients can be determined as

(19) $$\left\{\begin{array}{c} a_{i}={\tilde{y}}_{i-1},\;\\ b_{i}=\frac{{\tilde{y}}_{i}-{\tilde{y}}_{i-1}}{\delta_{i-1}}-\frac{k_{i}+2k_{i-1}}{6}\delta_{i-1},\;\\ c_{i}=\frac{k_{i-1}}{2},\;\\ d_{i}=\frac{k_{i}-k_{i-1}}{6\delta_{i-1}} \end{array}\right.$$

for i=1,2. Substituting (15) and (19) into the third spline condition in (18) and applying the natural boundary conditions [26,27], i.e., $p_{c,1}^{\prime \prime }\left(\tau_{0}\right)=p_{c,2}^{\prime \prime }\left(\tau_{2}\right)=0$, one can construct a system of linear equations

(20) Ak=b,

which provides a unique solution to unknown parameters $k_{i}$, i=0,1,2. Here,

(21) $$A=\left[\begin{array}{ccc} \;\;1 & 0 & 0\;\;\\ \;\;\delta_{0} & 2(\delta_{0}+\delta_{1}) & \delta_{1}\;\;\\ \;\;0 & 0 & 1\;\; \end{array}\right],$$

(22) $$b=6\left[\begin{array}{c} 0\;\\ \;\;\frac{{\tilde{y}}_{0}}{\delta_{0}}-{\tilde{y}}_{1}\frac{\delta_{0}+\delta_{1}}{\delta_{0}\delta_{1}}+\frac{{\tilde{y}}_{2}}{\delta_{1}}\;\;\;\\ 0 \end{array}\right],$$

and $k={\left[k_{0}\;k_{1}\;k_{2}\right]}^{T}$. Note that since the interpolation of complex SAR data is of the local type, where only three data samples are used, we assume that signal is compactly supported on $\left[\tau_{0},\tau_{2}\right]$ and employ the natural boundary conditions.

The resulting filter for estimating complex data value at range time $\tau_{p}$ based on cubic-spline interpolation can be constructed by employing (14) as

(23) $$p_{c,1}\left(\tau_{p}\right)=a_{1}+b_{1}\left(\tau_{p}-\tau_{0}\right)+c_{1}{\left(\tau_{p}-\tau_{0}\right)}^{2}+d_{1}{\left(\tau_{p}-\tau_{0}\right)}^{3},$$

where the complex-valued polynomial coefficients $a_{1}={\tilde{y}}_{0}$, $b_{1}=\left({\tilde{y}}_{1}-{\tilde{y}}_{0}\right)/\delta_{0}-\delta_{0}\left(k_{1}+2k_{0}\right)/6$, $c_{1}=k_{0}/2$, and $d_{1}=\left(k_{1}-k_{0}\right)/6\delta_{0}$ are obtained by substituting parameters *k* determined in (20) to (19) for i=1.

### 3.4. Extended Sinc Interpolation

Sinc interpolation is the method that is used in the reconstruction of bandlimited signals. Assume that the signal bandwidth is known and the signal is sampled at the rate $f_{s}$, which is two times or more higher than the maximal frequency in the considered frequency band $f_{\max}$, i.e., $f_{s}\geq 2f_{\max}$. Then, by employing the sampling theorem [28], the signal can be perfectly reconstructed from its samples by employing the following relation

(24) $$y\left(\tau \right)=\sum_{i=-\infty }^{\infty }y_{i}\mathrm{sinc}\left[\frac{\pi (\tau -\tau_{i})}{T_{s}}\right],$$

where $y_{i}=y\left(iT_{s}\right)$ and $\tau_{i}=iT_{s}$. Here, the interpolation kernel is represented by normalized sinc functions and $T_{s}$ is the sampling time. However, since the sinc function is infinite, it is difficult to implement an ideal reconstruction of a sampled signal via (24). Furthermore, the problem becomes even more complicated, when signal is complex-valued and phase control under reconstruction procedure is required.

Assume that the range time $\tau_{p}$ defined in (4) satisfies the condition $\tau_{0}<\tau_{p}<\tau_{1}$, which is similar to the case for linear and cubic interpolations. To implement the reconstruction procedure, the normalized sinc kernel presented in (24) can be truncated up to a finite number of sinc functions 2L+1, where *L* is a nonnegative integer, i.e., L∈Z and L≥0. The functions are equally translated in both directions from the interpolation point and normalized with the sampling time $T_{s}$ to reach zero value at known range-time samples $\tau_{i}$. Then, the resulting filter for data estimation at range time $\tau_{p}$ based on sinc interpolation gets the form

(25) $$y\left(\tau_{p}\right)=\sum_{i=-L}^{L}{\tilde{y}}_{i}w_{i}\mathrm{sinc}\left[\frac{\pi (\tau_{p}-\tau_{i})}{T_{s}}\right],$$

where ${\tilde{y}}_{i}$ are the reference data samples, the phase of which is controlled through the procedure (6) and may vary with a factor of π subject to their corresponding location in the complex plane, i.e., in the right $C_{+}$ or in the left complex half-plane $C_{-}$. Furthermore here, $w_{i}$ denotes the Hanning window, which is given by

(26) $$w_{i}=0.5+0.5\cos\left(\frac{\pi i}{L}\right),\;-L\leq i\leq L,$$

for i∈Z and used to suppress the Gibbs phenomenon in the truncated normalized sinc kernel [22].

## 4. Simulations

In this section, a simulation-based imaging scenario that is performed for a point scatterer in the frequency range [0.22,0.33]THz is considered. The simulations are performed to identify the most appropriate interpolation method as well as the optimal number of functions needed for the truncated sinc kernel. The analytical approach, which is described in Appendix A, and the peak-sidelobe ratio are used to validate the accuracy of the considered interpolation methods. All the simulations and analytical calculations have been carried out in MATLAB software.

### 4.1. Simulation Setup

Consider a point target placed at the reference range distance $R_{0}=2\;m$ from a quadcopter-based THz SAR imaging system, similarly as depicted in Figure 1. Assume for simplicity that the SAR system transmits a frequency-modulated continuous signal of the form

(27) $$s_{t}\left(\tau \right)=e^{j2\pi f_{c}\tau +j\pi \frac{f_{\max}-f_{\min}}{T_{p}}\tau^{2}},\;-\frac{T_{p}}{2}\leq \tau \leq \frac{T_{p}}{2},$$

with a duration $T_{p}=0.1\mu s$, and receives backscattered echoes of a similar waveform

(28) $$s_{r}\left(\xi ,\tau \right)=A_{t1}\mathrm{rect}\;\left\{\frac{\tau -\tau_{t1}}{T_{p}}\right\}e^{j2\pi f_{c}\left(\tau -\tau_{t1}\right)+j\pi \frac{f_{\max}-f_{\min}}{T_{p}}{\left(\tau -\tau_{t1}\right)}^{2}},$$

which is a reduced version of (3) for j=1. The range compressed received signal is given by

(29) $$g\left(\xi ,\tau \right)=A_{t1}\mathrm{sinc}\left[\pi \left(f_{\max}-f_{\min}\right)\left(\tau -\tau_{t1}\right)\right]e^{j2\pi f_{c}\left(\tau -\tau_{t1}\right)}$$

for $T_{p}\gg \tau -\tau_{t1}$. Note that the range-compressed version of backscattered echoes in (29) agrees with the time-domain representation of raw data obtained in (5). Here, the center frequency $f_{c}=0.275\;\mathrm{THz}$, $f_{\min}=0.22\;\mathrm{THz}$, $f_{\max}=0.33\;\mathrm{THz}$, and the two range time $\tau_{t1}=2R/c_{0}$, where *R* can be determined by (1). The system parameters are summarized in Table 1.

### 4.2. Results

**Figure 2:**
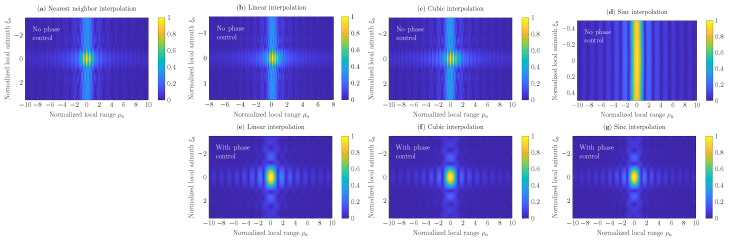
Reconstructed SAR scenes *h* of 251×251 pixels with nearest neighbor, linear, cubic, and sinc (for L=12) interpolations. Here, the sampling rate $f_{s}=f_{\max}=0.33\;\mathrm{THz}$.

In Figure 2 is shown SAR images $h(\xi_{n},\rho_{n})$ of 251×251 pixels reconstructed with the GBP algorithm (2) for sampling rate $f_{s}=f_{\max}=0.33\;\mathrm{THz}$. Here, the intensity is normalized with the peak intensity value, the range ρ and the azimuth ξ are normalized with −3dB beamwidths. The reconstruction procedure has been performed by corresponding incorporation of the nearest neighbor (7), linear (13), cubic (23), and sinc (25) interpolation algorithms to GBP. To evaluate the efficiency of the proposed interpolation algorithms, SAR scenes reconstructed at the rate $f_{s}=f_{\max}$ based on conventional linear, cubic, and sinc interpolators (i.e., those, in which the phase-control procedure is *not included*), are depicted in Figure 2b–d, respectively. The results demonstrate that linear, cubic, and sinc interpolators provide more accurate results at the Nyquist rate $f_{s}=f_{\max}$ in comparison with the nearest neighbor approach and corresponding conventional interpolators, where distortions over the normalized range interval $\rho_{n}\in \left[-2,2\right]$ are observed; see Figure 2a–g. Furthermore, sinc interpolation without the phase-control procedure provides a defocused reconstruction of the point target along the azimuth axis, as depicted in Figure 2d. It has been investigated that the involvement of the phase control procedure (6) increases the computational costs of linear, cubic, and sinc interpolators additionally by 14, 21, and 7(2L+1) operations per each iteration of the GBP algorithm, respectively, where *L* is the summation limit in the truncated sinc kernel of the extended sinc interpolator. However, the order of computational complexity of the GBP algorithm remains the same, i.e., $O\{N_{\xi }M_{\rho }M_{\xi }\}$, where $N_{\xi }=345$ denotes the number of aperture positions, $M_{\rho }=M_{\xi }=251$ the number of SAR-image pixels in range and azimuth directions, respectively.

**Figure 3:**
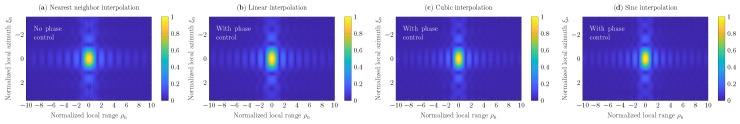
Reconstructed SAR scenes *h* of 251×251 pixels with nearest neighbor, linear, cubic, and sinc (for L=12) interpolations. Here, the sampling rate $f_{s}=2f_{\max}=0.66\;\mathrm{THz}$.

When the sampling rate is twice higher, i.e., $f_{s}=2f_{\max}$, the point target can be reconstructed accurately with all the interpolation methods discussed in this paper, as shown in Figure 3a–d. However, the azimuthal and some minor range distortions are still observed over the normalized range interval $\rho_{n}\in \left[-2,2\right]$, when the nearest neighbor approach is used; see Figure 3a. Note that the upsampling procedure for the nearest neighbor interpolator can be provided through the FFT interpolation, the computational complexity which is $O\{2N_{\xi }uN_{\rho }\log_{2}\left\{uN_{\rho }\right\}\}$, where u≥2, u∈Z, is the upsampling factor, $N_{\rho }=66017$ the number of range samples. It has been investigated that for upsampling factors u≥2, the computational complexity of the FFT interpolation becomes dominant over the computational complexity of the global backprojection algorithm. This fact demonstrates additional advantages of the extended interpolators, which provide the opportunity to avoid the FFT-based upsampling procedure, in terms of computational costs and memory resources. It should also be noted that the kernel of the sinc interpolator used in this numerical example is truncated to 2L+1=25 normalized sinc functions, where L=12 and based on which the reconstruction results are accurate; see Figure 2g and Figure 3d. Nevertheless, the optimal length of the kernel has to be investigated.

**Figure 4:**
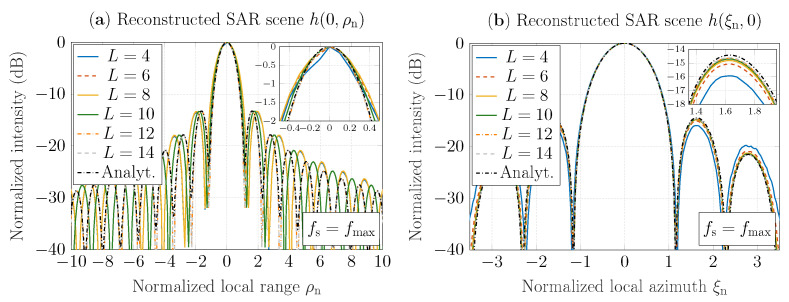
Evaluation of truncated normalized sinc kernel based on reconstructed SAR scenes $h(\xi_{n},\rho_{n})$ for the sampling rate $f_{s}=f_{\max}=0.33\;\mathrm{THz}$: (**a**) for $\xi_{n}=0$; (**b**) for $\rho_{n}=0$.

Figure 4a,b depict SAR-scene cuts reconstructed by the GBP algorithm that involves sinc interpolation, as well as SAR-scene cuts obtained based on the impulse response function (A1), which is defined in Appendix A, for φ=0 and φ=π/2, respectively. The scene cuts are plotted as functions of the normalized range $\rho_{n}$ (for $\xi_{n}=0$) and the normalized azimuth $\xi_{n}$ (for $\rho_{n}=0$), respectively, where the intensity is normalized with the peak intensity value. Here, the sampling rate $f_{s}=f_{\max}=0.33\;\mathrm{THz}$, the range and the azimuth are normalized similarly as in the example described above, and the kernel is truncated to the length 2L+1, where L∈{4,6,8,10,12,14}. It has been observed that for L≥12, the reconstruction results converge both for range and azimuth SAR-scene cuts. Furthermore, the results have a good agreement with the solution based on the approach (A1) in range and azimuth directions. It should be noted that intensity deviation in the third sidelobe, which is observed in Figure 4b, can be reduced by increasing the sampling rate $f_{s}$. Hence, the sinc interpolator with the kernel truncated up to 2L+1=25 normalized sinc functions, where L=12, is sufficient to obtain accurate interpolation results. Therefore, let the truncated sinc interpolator (25) for L=12 be defined as the *sinc interpolator* and used in the rest of the paper.

**Figure 5:**
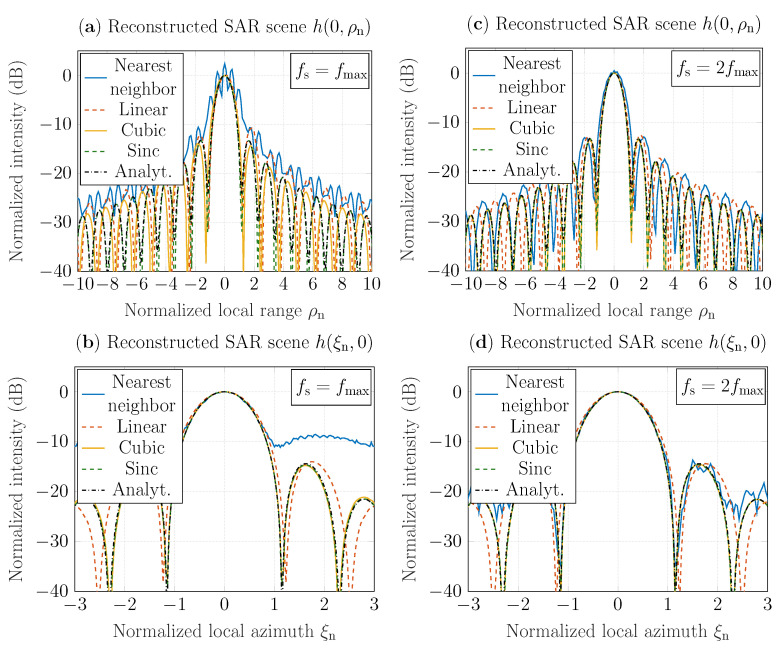
Evaluation of interpolation methods based on reconstructed SAR scenes of a point target $h(\xi_{n},\rho_{n})$ for the sampling rate: (**a**,**b**) $f_{s}=f_{\max}=0.33\;\mathrm{THz}$; (**c**,**d**) $f_{s}=2f_{\max}=0.66\;\mathrm{THz}$.

In Figure 5a,b are shown SAR-scene cuts $h(\xi_{n},\rho_{n})$ that are reconstructed for the Nyquist rate based on the GBP algorithm and the interpolation methods described in Section 3 and plotted as functions of normalized range (for $\xi_{n}=0$) and normalized azimuth (for $\rho_{n}=0$). Here, the reconstructed SAR-scene cuts are compared the results provided by the impulse response function (A1) for φ=0 and φ=π/2. Here, the intensity of cuts is normalized with the intensity value of the SAR-scene center, i.e., with h(0,0). Furthermore, range and azimuth are normalized similarly to the examples described above. It has been observed that reconstructions obtained with the cubic and sinc interpolation techniques are more accurate both in range and azimuth directions than the results obtained based on the nearest neighbor and linear interpolation algorithms. The results based on nearest neighbor interpolation contain strong distortions in range and indistinguishable sidelobes in azimuth. The reconstructions based on cubic and sinc interpolations have the same azimuth resolution as the analytically-based result (A1); see Figure 5b. However, in comparison with the SAR-scene cuts based on sinc interpolation and (A1), $h(0,\rho_{n})$ based on the cubic interpolation algorithm has lower range resolution; see Figure 5a. It has also been investigated that sinc interpolator provides the most accurate reconstruction results in terms of the peak-sidelobe ratio (PSLR) (A4), which is introduced in Appendix B, and the root mean square error (RMSE). The PSLR-deviation of SAR scene *h*, which is reconstructed with sinc interpolation, from the analytical solution at the Nyquist rate is around 0.5%; see the calculated PSLRs in Table 2. It should be noted that PSLR of *h* obtained with the extended linear interpolation procedure is higher (18.9%-deviation from the analytical PSLR) in comparison with the ratios of scenes obtained with the extended cubic and sinc interpolation approaches. To determine RMSE, we used the results obtained via the impulse response function (A1) as reference values; the results are summarized in Table 3. The results for SAR scene cuts in the range and azimuth directions demonstrate that the extended sinc interpolator provides the most accurate results at the Nyquist rate with an error around 0.7%. Note that linear and cubic interpolators provide good accuracy with deviation from the reference results around 3% and 1.26%, respectively, in range, and around 1.18% and 0.79% in azimuth, respectively. In the case of the nearest neighbor interpolator, the upsampling procedure is required.

Figure 5c,d depict reconstructed cuts $h(\xi_{n},\rho_{n})$ plotted and compared similarly as in Figure 5a,b, but for the twice increased sampling rate, i.e., for $f_{s}=2f_{\max}$. The reconstruction accuracy based on the nearest neighbor interpolation improved significantly by increasing the sampling rate, with the RMSE reduced from 24% to 6.4%; see Table 3. However, the reconstruction result does not agree with the results provided by linear interpolator in the range direction. It contains sidelobe distortions in azimuth SAR-scene cut, as shown in Figure 5c,d. Furthermore, the SAR-scene cuts $h(0,\rho_{n})$ and $h(\xi_{n},0)$ that are reconstructed with the nearest neighbor and linear approaches still have a lower range and azimuthal resolution, which can be improved with a further increase of the sampling rate. It has also been investigated that by increasing the sampling rate, the SAR-scene cuts obtained with cubic interpolator have the same spatial resolution as the result based on the impulse response function (A1). Furthermore, the reconstructed scene *h* based on cubic interpolation deviates from the analytical result around 2.4% in terms of PSLR and less than 0.8% in terms of RMSE; see PSLRs in Table 2 and RMSE in Table 3, respectively. Hence, it can be concluded that the incorporation of cubic and sinc interpolators into the GBP algorithm provides the most accurate reconstruction of SAR scenes, which motivates further investigations with application to real data.

## 5. Experimentation

**Figure 6:**
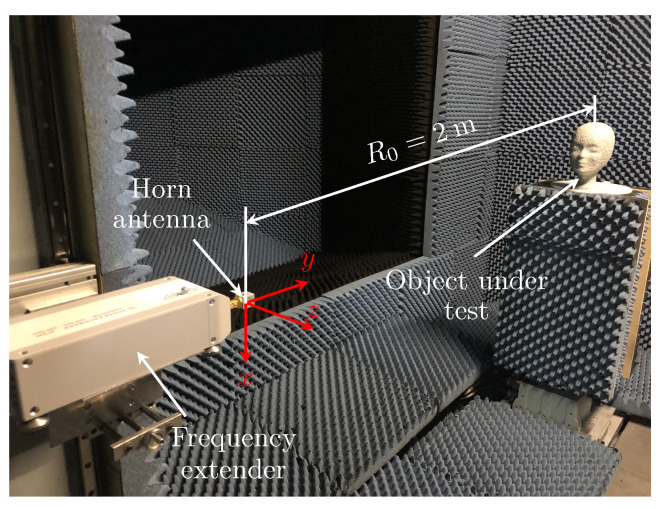
Measurement setup. A ground-based monostatic THz SAR imaging system was based on a vector network analyzer (VNA) and a transceiver, which was mounted on a mobile platform. The transceiver consisted of a frequency extender and a horn antenna to perform measurements in the frequency range [0.22, 0.33] THz. Here, $R_{0}$ denotes the reference range distance between the platform antenna and the object under test (mannequin head).

In this section, we describe imaging of a mannequin head that was performed in the frequency range [0.22, 0.33] THz and validate the efficiency of the developed interpolation approaches on the real data. The head is made of a foam material and coated with metallic paint. The measurement data was acquired via the setup depicted in Figure 6.

### 5.1. Measurement Setup

The monostatic THz SAR imaging system was based on Rohde&Schwarz ZVA67 VNA, the operating frequency range of which is between 10MHz and 67GHz. The setup was further equipped with Rohde&Schwarz ZC330 frequency extender and the rectangular horn antenna, the parameters of which are described in ([13] Table 1, p. 578), which gave the opportunity to perform one-port measurements of the reflection coefficient in the frequency range [0.22, 0.33] THz. Note that the reflection coefficient measured in the frequency domain can then be transformed to the time domain and used for the SAR scene reconstruction. The frequency extender incorporated with the horn antenna as a transceiver was mounted on the mobile platform, which was shifted in the azimuthal (vertical) direction with a uniform step $\Delta_{\xi }=1\;\mathrm{mm}$ to form a synthetic aperture. The object under test was placed at the reference range distance $R_{0}=2\;m$ from the platform antenna. The measurements were performed based on stop-and-go approximation: at each corresponding measurement position, a frequency sweep was transmitted and received, respectively. The total number of measurement and frequency points was 344 and 3001, respectively. The acquired raw data g(ξ,τ) was filtered in the frequency domain with the cosine-tapered window (the cosine fraction α=0.25) and time-gated [29] to suppress from the surrounding environment, outside of the range of interest [30]. The measurement setup parameters are summarized in Table 4.

### 5.2. Results

**Figure 7:**
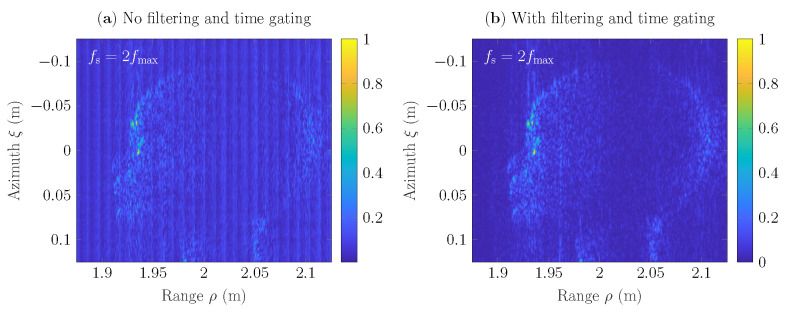
SAR scenes *h* of 251×251 pixels reconstructed with nearest neighbor interpolation for the sampling rate $f_{s}=2f_{\max}=0.66\;\mathrm{THz}$. Here, the raw data g(ξ,τ): (**a**) has not been postprocessed; (**b**) has been filtered in the frequency domain and time gated.

In Figure 7 is shown comparison of SAR scenes h(ξ,ρ) of 251×251 pixels that have been reconstructed from the acquired raw data g(ξ,τ). Here, nearest neighbor interpolation algorithm (7) has been used as a part of GBP to reconstruct SAR scenes, and the sampling rate is $f_{s}=2f_{\max}=0.66\;\mathrm{THz}$. Figure 7a, depicts the result, where the raw data has not been filtered and time-gated. The scene contains noise, which is caused by reflections of the surrounding environment, as well as by the absence of the phase control procedure in the nearest neighbor interpolation algorithm. In Figure 7b, the SAR scene has been reconstructed from the raw data, which has been postprocessed, i.e., filtered in the frequency domain and time-gated. It has been observed that the postprocessing procedure allows us to suppress ringing noise in the SAR scene and improves the visibility of the object under test. Nevertheless, the SAR scene in Figure 7b contains distortions, which requires the use of other interpolation methods to suppress them and, furthermore, an investigation of the appropriate sampling rate. Thus, in the rest of the examples, the acquired raw data will be filtered in the frequency domain and time-gated.

**Figure 8:**
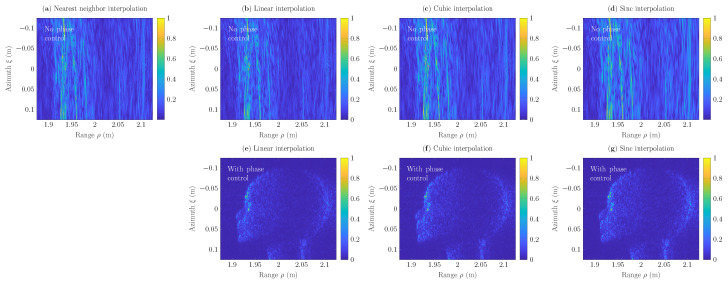
Reconstructed SAR scenes *h* of 251×251 pixels with nearest neighbor, linear, cubic, and sinc interpolations: (**a**–**d**) without the phase-control procedure; (**e**–**g**) with the phase control procedure. Here, the sampling rate $f_{s}=f_{\max}=0.33\;\mathrm{THz}$.

Figure 8 depicts scenes h(ξ,ρ) of 251×251 pixels reconstructed at the Nyquist sampling rate via the GBP algorithm (2), into which nearest neighbor (7), linear (13), cubic (23), and sinc (25) interpolation algorithms have been incorporated, respectively. Furthermore, SAR-scenes reconstructed at the Nyquist rate based on conventional linear, cubic, and sinc interpolators are included for comparison; see Figure 8b–d, respectively. Here, the intensity of SAR scenes is normalized with the peak intensity, similarly as in Figure 2. It has been observed that linear, cubic, and sinc interpolators provide an accurate reconstruction of the mannequin head at the Nyquist rate in comparison with corresponding conventional sinc interpolators that do not include the phase-control procedure (6). When nearest neighbor interpolation is used, strong azimuthal distortions occur and the image cannot be reconstructed accurately. To suppress them, the sampling rate has to be increased.

**Figure 9:**
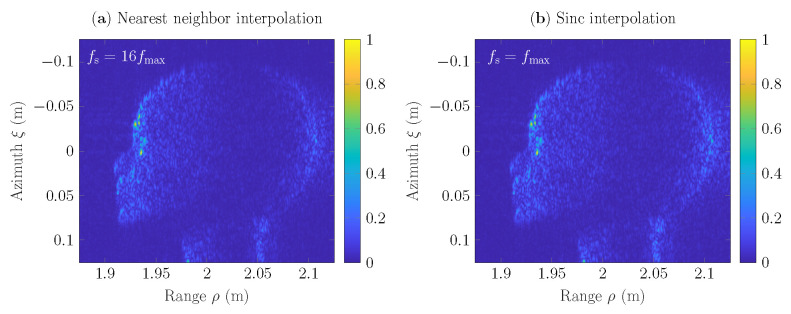
SAR scenes *h* of 251×251 pixels reconstructed with: (**a**) nearest neighbor interpolation for the sampling rate $f_{s}=16f_{\max}=5.28\;\mathrm{THz}$; (**b**) sinc interpolation for the sampling rate $f_{s}=f_{\max}=0.33\;\mathrm{THz}$.

In Figure 9 is depicted the comparison of SAR scenes h(ξ,ρ), the reconstruction of which has been based on the incorporation of nearest neighbor and sinc interpolators into the GBP algorithm. Here, the results based on the nearest neighbor approach are given for the rate $f_{s}=16f_{\max}=5.28\;\mathrm{THz}$ and provide approximately similar accuracy, as the results obtained via the sinc interpolation at the Nyquist rate $f_{s}=f_{\max}=0.33\;\mathrm{THz}$. The comparison demonstrates that to obtain an accurate reconstruction of the object under test, even at the Nyquist rate, it is enough to employ one of the proposed extended interpolation algorithms that contain the phase control procedure (6) into the GBP algorithm (2).

## 6. Conclusions

In this paper, the existing linear, cubic, and sinc interpolation algorithms have been extended and adapted to process complex SAR data. The extended algorithms include the phase control procedure that relates the phase value of interpolated complex SAR data to the given range-time sample. The proposed phase control procedure (6) has increased computational costs of linear, cubic, and sinc interpolators additionally by 14, 21, and 7(2L+1) operations per each iteration of the global backprojection algorithm, respectively, where *L* denotes the summation limit in the truncated sinc kernel of the extended sinc interpolator. The developed interpolation approaches are incorporated into the GBP algorithm, and their efficiency is tested at THz frequencies. Similarly, the interpolation methods can be incorporated into other backprojection algorithms, such as fast and fast factorized backprojections. It can be concluded that the use of the phase control procedure (6) provides the opportunity to achieve accurate image reconstruction results both in azimuth and range directions, even at the Nyquist sampling rate. At the same time, to reach an approximately similar level of reconstruction accuracy with the nearest neighbor approach, sixteen times higher sampling rate, $f_{s}=16f_{\max}$, is required. It has been investigated that for such a sampling rate and at THz frequencies, the computational complexity of the nearest neighbor interpolator becomes much higher than the computational complexity of the extended interpolation algorithms at the Nyquist rate. Correspondingly, the amount of raw data needed in the image reconstruction procedure can be significantly reduced, which is of great importance in the SAR hardware realization, especially at THz frequencies.

## Figures and Tables

**Table 1:** Simulation Setup Parameters.

Parameter	Value
The highest frequency processed, $f_{\max}$	0.33THz
The lowest frequency processed, $f_{\min}$	0.22THz
Number of aperture positions, $N_{\xi }$	345
Aperture step, $\Delta_{\xi }$	0.997mm
Integration angle, $\phi_{0}$	≈$9.8^{\circ }$
Reference range, $R_{0}$	2m

**Table 2:** Peak-sidelobe Ratios (in dB).

	Sampling Rate	$f_{\max}$	$2f_{\max}$
Interpolation			
Nearest neighbor		−7.395	−13.37
Linear		−10.756	−12.555
Cubic		−14.372	−13.586
Sinc		−13.335	−13.331
Analytical		−13.265	

**Table 3:** Root Mean Square Error (in %).

	Sampling Rate	$f_{\max}$		$2f_{\max}$	
Interpolation					
Nearest neighbor		12.92	23.93	6.36	2.3
Linear		3.02	1.18	1.02	0.79
Cubic		1.26	0.79	0.77	0.71
Sinc		0.71	0.72	0.71	0.71

**Table 4:** Measurement Setup Parameters.

Parameter	Value
The highest frequency processed, $f_{\max}$	0.33THz
The lowest frequency processed, $f_{\min}$	0.22THz
Number of aperture positions, $N_{\xi }$	344
Number of frequency bins, $N_{f}$	3001
Aperture step, $\Delta_{\xi }$	1mm
Integration angle, $\phi_{0}$	≈$9.8^{\circ }$
Reference range, $R_{0}$	2m

## Data Availability

The data presented in this study are available on request from Aman Batra.

## Appendix A. Impulse Response Function

A SAR scene *h* that contains a point target located in its center can be expressed analytically as an impulse response function in polar coordinates ([4], Equations (9)–(11))

(A1) $$h\left(\varrho ,\varphi \right)=\frac{e^{j\varphi }}{\varrho }h_{s}\left(\varrho ,\varphi \right)+\phi_{0}\frac{e^{j\varphi }}{\varrho }\sum_{n=-\infty }^{\infty }\frac{j^{n}h_{j,n-1}\left(\varrho \right)}{e^{j(n-1)\varphi }}\mathrm{sinc}\left(n\frac{\phi_{0}}{2}\right),$$

where

(A2) $$\begin{array}{c} \;h_{s}\left(\varrho ,\varphi \right)=B_{r}\mathrm{sinc}\left[\frac{B_{r}}{2}\varrho \cos\left(\frac{\phi_{0}}{2}-\varphi \right)\right]e^{j\varrho \cos\left(\frac{\phi_{0}}{2}-\varphi \right)+j\frac{\phi_{0}}{2}}\\ \;\;\;\;\;\;\;\;\;\;\;\;\;\;-B_{r}\mathrm{sinc}\left[\frac{B_{r}}{2}\varrho \cos\left(\frac{\phi_{0}}{2}+\varphi \right)\right]e^{j\varrho \cos\left(\frac{\phi_{0}}{2}+\varphi \right)-j\frac{\phi_{0}}{2}} \end{array}$$

and

(A3) $$h_{j,n-1}\left(\varrho \right)=\left(1-\frac{B_{r}}{2}\right)J_{n-1}\left[\left(1-\frac{B_{r}}{2}\right)\varrho \right]-\left(1+\frac{B_{r}}{2}\right)J_{n-1}\left[\left(1+\frac{B_{r}}{2}\right)\varrho \right].$$

Here, ϱ and φ are related to azimuth and range via ξ=ϱsinφ and ρ=ϱcosφ, respectively, $J_{n-1}\left(\cdot \right)$ is the Bessel function of the first kind [31], $B_{r}=B/f_{c}$ the relative bandwidth, and $\phi_{0}$ the integration angle. Note that analytical realizations of SAR-scene cuts h(0,ρ) and h(ξ,0) can be obtained from the impulse response function (A1) for φ=0 and φ=π/2, respectively.

## Appendix B. Peak-Sidelobe Ratio

The image quality of a reconstructed SAR scene *h* can be evaluated in terms peak-sidelobe ratio [32]. The peak-sidelobe ratio (PSLR) is a dimension that characterizes the ability to measure weak reflective targets in the presence of strong reflective targets in their neighborhood. PSLR can mathematically be expressed in dB as

(A4) $${\mathrm{PSLR}|}_{\mathrm{dB}}=10\log_{10}|I_{\mathrm{SL}}|-10\log_{10}\left|I_{\mathrm{ML}}\right|,$$

where $I_{\mathrm{SL}}$ and $I_{\mathrm{ML}}$ denote the peak-intensity value in the sidelobe and in the main-lobe area, respectively.
